# Clinical predictors of elective total joint replacement in persons with end-stage knee osteoarthritis

**DOI:** 10.1186/1471-2474-11-86

**Published:** 2010-05-06

**Authors:** Joseph A Zeni, Michael J Axe, Lynn Snyder-Mackler

**Affiliations:** 1 Department of Physical Therapy, University of Delaware, Newark, DE, USA; 2 First State Orthopaedics, Newark, DE, USA

## Abstract

**Background:**

Arthritis is a leading cause of disability in the United States. Total knee arthroplasty (TKA) has become the gold standard to manage the pain and disability associated with knee osteoarthritis (OA). Although more than 400 000 primary TKA surgeries are performed each year in the United States, not all individuals with knee OA elect to undergo the procedure. No clear consensus exists on criteria to determine who should undergo TKA. The purpose of this study was to determine which clinical factors will predict the decision to undergo TKA in individuals with end-stage knee OA. Knowledge of these factors will aid in clinical decision making for the timing of TKA.

**Methods:**

Functional data from one hundred twenty persons with end-stage knee OA were obtained through a database. All of the individuals complained of knee pain during daily activities and had radiographic evidence of OA. Functional and clinical tests, collectively referred to as the Delaware Osteoarthritis Profile, were completed by a physical therapist. This profile consisted of measuring height, weight, quadriceps strength and active knee range of motion, while functional mobility was assessed using the Timed Up and Go (TUG) test and the Stair Climbing Task (SCT). Self-perceived functional ability was measured using the activities of daily living subscale of the Knee Outcome Survey (KOS-ADLS). A logistic regression model was used to identify variables predictive of TKA use.

**Results:**

Forty subjects (33%) underwent TKA within two years of evaluation. These subjects were significantly older and had significantly slower TUG and SCT times (p < 0.05). Persons that underwent TKA were also significantly weaker, had lower self-reported function and had less knee extension than persons who did not undergo TKA. No differences between groups were seen for BMI, gender, knee flexion ROM and unilateral versus bilateral joint disease. Using backward regression, age, knee extension ROM and KOS-ADLS together significantly predicted whether or not a person would undergo TKA (p ≤ 0.001, R^2 ^= 0.403).

**Conclusions:**

Younger patients with full knee ROM who have a higher self-perception of function are less likely to undergo TKA. Physicians and clinicians should be aware that potentially modifiable factors, such as knee ROM can be addressed to potentially postpone the need for TKA.

## Background

Osteoarthritis (OA) is a degenerative condition that affects millions of persons in the United States. The knee is the most commonly affected joint and knee OA is a leading cause of disability and functional limitations in adults [1,2]. The most common surgical intervention for end-stage knee OA is total knee arthroplasty (TKA). Although more than 400,000 primary TKA surgeries are performed each year in the United States, not all individuals with knee OA elect to undergo the procedure [3-5]. No clear consensus exists on criteria to determine who should undergo TKA, however, severe pain, higher levels of disability and excessive cartilage degeneration appear to be decisive factors for the procedure [6,7]. Currently, no longitudinal studies have evaluated clinical impairments that precipitate the use of TKA in persons with knee OA.

Determinants of the decision to perform TKA have mainly been derived from surveys of orthopedic surgeons [8]. Surgeons are less likely to prescribe total joint replacement in the presence of cardiovascular or psychological co-morbidities and are more likely to perform total joint replacements on individuals with severe pain, those with radiographic evidence of end-stage cartilage degeneration and for men [4,8-11]. These surveys provide important insight into the rationale of prescribing surgery, but they do not reflect the motivations of the patient's decision to undergo TKA. Previous studies that have included a longitudinal assessment of predictive factors from a patient's perspective have utilized a questionnaire-based format, which only permits self-perceived assessment of functional ability [12,13]. Self-perceived performance often substantially differs from an individual's actual functional capabilities [14,15].

Demographic and socioeconomic differences on TKA use have been reported, although the evidence is conflicting. The majority of these investigations have reported that women are less likely to undergo TKA, despite a higher prevalence of OA and worse symptoms [16,17]. This may be related to the fact that surgeons are more likely to recommend TKA for males [10,11]. Race has been found to be a non-modifiable risk factor for undergoing TKA, with Caucasian individuals being 1.5 times more likely to undergo TKA than non-white individuals [18]. Hispanic and black persons show a propensity to not utilize TKA, even when accounting for differences in economic access and health needs [19].

In addition to demographic differences, the rate of cartilage loss and radiographic severity of OA are indicative of future joint replacement [20-22]. Cartilage loss measured by radiographic joint space narrowing is the gold standard for quantifying the mechanical changes associated with disease progression. Despite this, radiographic changes do not necessarily correlate to symptoms and functional impairments [23]. It is therefore more likely that an assessment of structural changes, functional ability and pain are collectively needed to accurately predict who will require TKA [7,24].

Knowledge of the underlying clinical motivations of persons undergoing TKA is essential, as pre-operative factors affect post-operative outcomes. Lower quadriceps strength, increased pain and decreased knee flexion have been shown to result in lower post-operative outcomes [25,26]. If these are the same clinical predictors that influence the decision to undergo TKA, then patients should be educated to the benefits of undergoing TKA prior to the onset of these severe limitations. Adequate patient education has the potential to reduce the time and cost associated with post-operative physical therapy, while improving patient satisfaction.

A considerable time commitment is required when opting to undergo TKA. Significant time off from work, out-of-pocket expense and physical assistance may be required for the surgical procedure, as well as the recovery and rehabilitation processes [27]. Patients may find it beneficial to delay the surgery until they are able to make the financial and time commitment or able to coordinate post-operative care. To do so, it is imperative that clinicians understand the functional limitations that necessitate TKA intervention. If the limitations are treatable with preventative or palliative measures, then therapeutic modalities aimed at reducing these limitations could successfully delay the surgery.

Currently, a longitudinal analysis of objective clinical measures that predict the decision to undergo TKA is lacking in the literature. The purpose of this study was to determine which clinical factors will predict the decision to undergo TKA in individuals with end-stage knee OA. Knowledge of these factors will aid in clinical decision making for the timing of TKA and lead to improved patient education, which may positively affect post-operative outcomes.

## Methods

Functional data from one hundred twenty persons (59 males, 61 females; mean age 60 years, SD 9.7, range 28-83) with end-stage knee OA were obtained through a database maintained by the University of Delaware Physical Therapy Clinic and approved by the Human Subjects Review Board. All subjects were referred to the physical therapy clinic from a single orthopedic surgeon who does not perform TKA. All of the individuals complained of knee pain during daily activities that prompted them to consult the orthopedic surgeon. Additionally, all subjects had radiographic evidence of OA in more than one compartment as evidenced by Kellgren-Lawrence scores ≥ 3 [28]. These criteria established our operational definition of end-stage knee OA. Functional evaluations were completed by a physical therapist and consisted of measuring height, weight, quadriceps strength, active knee range of motion, self-perceived functional ability, functional mobility and the ability to negotiate stairs. The functional evaluation, known as the Delaware Osteoarthritis Profile, assesses self-perceived functional ability, objective functional capabilities, range of motion, quadriceps strength and anthropometric measures. Only subjects who were definitively known to undergo or not undergo TKA were included in the analysis.

### Quadriceps Strength

For the purposes of this study, quadriceps strength was defined as the volitional force produced during unilateral isometric knee extension. Subjects were seated in a Kin-Com dynamometer (Harrison, TN, USA) with the knees flexed to ~75 degrees and hips flexed to ~90 degrees. Straps were placed across the thigh and the shank to prevent excessive movement during the isometric contraction. Subjects completed a warm-up trial and then two subsequent maximal isometric trials. Peak force from the two trials were averaged and normalized to the individual's body mass index (BMI). This method has been used to effectively measure strength in persons with knee OA [29].

### Knee Range of Motion

Active knee flexion and extension range of motion were collected in a supine position using a standard long arm goniometer. The axis of the goniometer was placed over the lateral femoral epicondyle. The proximal end of the goniometer was aligned with the greater trochanter of the femur and the distal arm was aligned with the lateral malleolus. Measurements were recorded with respect to full extension being 0 degrees with positive numbers indicating a more flexed position and negative numbers indicating hyperextension. During knee extension, the subject's heel was placed on an elevated block to allow clearance of the thigh and calf. Subjects were asked to maximally extend the knee and the average active knee extension ROM over two trials was recorded. During knee flexion, subjects were instructed to maximally flex the hip and knee and draw the heel toward the buttocks. The average peak knee flexion was recorded from two trials. Goniometric knee flexion and extension measurements are highly reliable in persons with knee OA [30].

### Knee Outcome Survey - Activities of Daily Living Subscale

The Knee Outcome Survey - Activities of Daily Living Subscale (KOS-ADLS) is a subsection of the Knee Outcome Survey that contains fourteen questions about an individual's perception of his or her ability to perform activities of daily living [31]. Answers to the questions are ranked from 0 (inability to perform activity) to 5 (no difficulty with activity). Answers are summed and given as a percentage score, with a higher score reflecting greater self-perceived functional ability. This is a reliable and valid test to use for individuals with knee pathology [32].

### Functional Tests

The Timed Up and Go (TUG) and Stair Climbing Task (SCT) were incorporated to assess functional mobility and stair negotiation. The TUG is a timed test in which the subjects rise from a chair, walk three meters, turn around and return to a seated position in the chair. The subjects performed two trials and the average time to complete the task was recorded. Subjects were permitted to use the arms of the chair during the rising from the chair and returning to a seated position. This test has excellent reliability and has been used to examine outcomes in persons with knee OA [33]. The SCT is also a timed test in which the subjects begin at the bottom of a flight of twelve stairs, ascend the steps on the investigators command, turn around and descend the stairs. Light handrail use was permitted for balance. These tests have previously been used to explore differences in persons with knee OA [26].

### Statistical Analysis

Independent t-tests were performed to determine if significant differences in age, BMI, TUG, SCT, KOS-ADLS, quadriceps strength on the involved and uninvolved side, active knee extension range of motion and active knee flexion range of motion existed between persons who did and persons who did not choose to undergo TKA within two years of evaluation. No adjustments were made for multiple comparisons. Chi-square tests were performed to determine if gender or unilateral versus bilateral joint disease were different between the two groups. Variables that were found to be significantly different between the two groups (p ≤ 0.05) were used as covariates in a logistic regression analysis. An exploratory backward regression model was created from this to determine if fewer variables could still explain a large portion of the variability in the data. Receiver operating characteristic curves were then created from these variables to determine clinically meaningful cut-off points. Missing data entries were ignored on a pair-wise basis and cases were not removed from the analysis secondary to an incomplete data set.

## Results

Forty subjects (33.3%; 18 males and 22 females; age range 46-78 years) underwent TKA within two years of evaluation. Mean time to TKA was 224 days following the physical therapy evaluation. These subjects were significantly older and had significantly slower TUG and SCT times (Table 1). Persons who underwent TKA were also significantly weaker in both the involved and uninvolved limb, had lower self-reported functional ability and had less knee extension than persons who did not undergo TKA. We found no differences between groups for BMI, gender, knee flexion ROM and unilateral versus bilateral joint disease.

**Table 1:** Mean (SD) of the baseline values and group differences in the subject sample.

	**Sample (n)**	**All Subjects**	**Had TKA (n = 40)**	**No TKA (n = 80)**	**p-value**
	
Age (years)	111	59.5 (9.7)	63.3 (8.3)	57.6 (9.8)	0.002*
Affected joint (Bi/Unilateral %)	120	44/56	46/54	43/57	0.845
Sex, M/F (%)	120	49/51	46/54	50/50	0.702
Body Mass Index (kg/m^2^)	115	32.2 (6.5)	32.5 (7.0)	32.0 (6.4)	0.740
TUG (s)	117	8.8 (2.5)	9.5 (2.3)	8.5 (2.5)	0.034*
SCT (s)	115	16.0 (8.8)	19.4 (10.8)	14.4 (7.0)	0.012*
KOS-ADLS (%)	99	58.2 (22.0)	51.1 (19.5)	61.5 (22.4)	0.027*
Quad Strength (Inv.) (N/BMI)	115	22.5 (11.4)	18.9 (9.1)	24.2 (12.0)	0.010*
Quad Strength (Uninv.) (N/BMI)	115	26.9 (11.1)	23.8 (9.4)	28.4 (11.5)	0.037*
Extension ROM (degrees)	120	1 (5)	3 (6)	0 (4)	0.013*
Flexion ROM (degrees)	120	125 (16)	124 (11)	125 (18)	0.671

Logistic regression revealed that age, KOS-ADLS, TUG, SCT, quadriceps strength and knee extension ROM significantly predicted TKA within two years (p ≤ 0.001, R^2 ^= 0.412). Younger age, higher KOS-ADLS scores, faster TUG and SCT times, stronger quadriceps and full knee extension predicted those who do not undergo TKA. Using backward regression, age, knee extension ROM and KOS-ADLS together significantly predicted whether or not a person would undergo TKA (p ≤ 0.001, R^2 ^= 0.403) (Table 2). Despite using only three variables in this model, there was only a small reduction in the R^2 ^value. Both regression models were better predictors of those who would not undergo TKA opposed to those who would undergo TKA.

**Table 2:** Model 1 of the logistic regression contained all of the significant variables.

Model	Variables Included	Correctly Predicted		Nagelkerke R^2^	Significance of Model
	Age, KOS-ADLS, TUG,	Had TKA	59%		
**Model 1**	SCT, Bilateral Strength,	No TKA	91%	0.412	<0.001
	Extension ROM	**Overall**	**81%**		

	Age, KOS-ADLS,	Had TKA	62%		
**Model 2**	Extension ROM	No TKA	86%	0.403	<0.001
		**Overall**	**78%**		

Model 2 contained the variables from the final backwards regression step.

Cut-off values based on maximizing the area under the receiver operating characteristic curve also did a better job predicting those who would not undergo TKA (Table 3). Odds ratio revealed that the risk of undergoing TKA increased by 1.13 times for every year increase in age (Table 3). For every degree of knee flexion contracture the chance of undergoing TKA increased by 1.23 times. Higher KOS-ADLS by one point reduced the chance of TKA by 1.04 times.

**Table 3:** Cut-off values from receiver operating characteristic and multivariate odds ratio from logistic regression analysis (eROM = extension range of motion).

	Odds Ratio	Had TKA	No TKA	Total	Percent Correct
	
Age (years)					
<= 60		16	48	64	75
>60	1.13	21	26	47	45
Total		37	74	111	
**eROM (degrees)**					
<= 0		15	50	65	77
>1	**1.23**	24	31	55	44
**Total**		**39**	**81**	**120**	

**KOS-ADLS (%)**					
>50	**0.96**	14	46	60	77
<= 50		18	21	39	46
**Total**		**32**	**67**	**99**	

## Discussion

Using the Delaware Osteoarthritis Profile, we have found a subset of objective clinical measures that predict whether persons with end-stage knee OA undergo elective TKA within two years of seeing an orthopedic surgeon for evaluation of knee pain. Reliable and valid clinical tests can be used to determine persons who will likely not undergo TKA within two years. Differences existed between those who had TKA and those who did not for a majority of the objective measures. This suggests that while radiographic severity may quantify end-stage disease, differences in clinical measures determine persons who will forgo TKA for greater than two years. The results of this study support recent findings by Dieppe et al., who concluded that simple measures of pain or radiographic evidence of OA do not encompass all of the clinical factors that determine who should undergo total joint arthroplasty [34].

We found no differences in the distribution of males and females between groups, suggesting that sex is not a factor related to the decision to undergo TKA. Similarly, bilateral versus unilateral disease and BMI were not significantly different between groups. This is an interesting finding because a higher BMI is strongly associated with the development of knee OA [35,36]. While BMI may be a risk factor for the initiation of the disease, our results do not support that BMI is related to the decision of whether to undergo TKA once subjects develop symptomatic end-stage OA.

Variables that were significantly different between groups explained a large portion of the variability in TKA use. Using only three clinical measures (age, knee extension ROM and KOS-ADLS scores) the model explained a large portion of the variability. The predictive ability of these factors was directional. Collectively, these factors correctly predicted nearly all of those who did not undergo TKA within 2 years (91% correct prediction) but only slightly more than half (58% correct prediction) of those who did. The receiver operator characteristic cutoffs had the same result, with a strong ability to predict those who did not undergo TKA. Individuals younger than 60 years old who have full knee extension ROM are much less likely to undergo TKA. Self-perceived functional ability greater than 50 as measured by the KOS-ADLS will further reduce the chances of undergoing TKA. The case for the opposite is not as strong and different factors not considered here may influence those who do choose to undergo TKA.

The odds ratio revealed that age, extension ROM and KOS-ADLS had a large increase in risk for undergoing TKA with a single unit increase in the variable. The mean difference between groups was not very large for knee extension. However, the odds ratio revealed that for a single degree increase in knee flexion contracture, the risk of undergoing TKA increased by 23%. This suggests small reductions in end-range knee motion may significantly increase one's chance of requiring TKA in the future. Clinicians and physicians should stress the importance of maintaining full ROM to patients presenting with end-stage knee OA. The efficacy of pre-operative rehabilitation regimens is often limited by knee pain. While aggressive strengthening protocols may not be feasible, perhaps patients wishing to delay surgery, who also present with knee flexion contracture, should participate in rehabilitation that focuses on maintaining full knee ROM. Loss of knee range of motion in other knee pathologies is suggested to expedite the progression of the disease. Following anterior cruciate ligament reconstruction, loss of knee extension range of motion may facilitate the development and progression of knee OA [37]. Knee flexion contractures were also associated with radiographic evidence of cartilage deterioration within 7 years of ACL reconstruction [38]. The results from the present study also suggest that knee flexion contracture is one of the most important factors for the progression of the disease.

Similar to knee extension ROM, age and KOS-ADLS also had relatively large odds ratios, highlighting the importance of these variables in the prediction of decision to undergo TKA. The mean difference in age was relatively large between the two groups, with persons undergoing TKA being an average of six years older. In addition, the chance of requiring TKA increased 13% for every year of age. Older age is the strongest predictor of radiographic joint space narrowing and the development of knee OA [35,39]. In this study, age is also a strong predictor of TKA use once symptomatic end-stage knee OA has developed, with younger persons less likely to undergo TKA. For every one percentage point decrease in KOS-ADLS score, the chances of undergoing TKA increased by 4%. While this may seem relatively small, the range of KOS scores was large. Therefore, a difference of ten to twenty points is not only clinically meaningful, but also dramatically increases the chance that one will undergo TKA. Age, knee extension ROM and self-perceived function are important variables that should be evaluated by clinicians when dispensing advice or influencing patients' expectations of TKA surgery.

Persons wishing to delay surgery should be referred to physical therapy to address age-related impairments, deficits in KOS-ADLS scores and loss of knee extension ROM. Deyle et al in a randomized control trial found that manual physical therapy in persons with knee OA delayed the need for TKA, which supports the findings in the present study [40]. Physical therapy regimens that include individualized manual therapy, supervised exercises and a home exercise component have also been shown to improve self-reported functional scores and functional ability in persons with knee OA [40-43]. Therefore, physical therapy may be the primary method of improving the variables that predicted the use of TKA, as it is effective in reducing pain and disability in people with OA [44,45].

One limitation of this study is that follow-up radiographs were not analyzed to estimate joint space narrowing prior to surgery, although all subjects demonstrated radiographic evidence of knee OA at their initial evaluation. Despite this, the functional measures explored in this study explain a large amount of the variability in people who do or do not undergo TKA within 2 years for end-stage knee OA. Since radiographic measures of OA severity do not strongly correlate to pain and disability, it is possible that using functional measures as determinants of future TKA are more appropriate [23,46]. This agrees with previous work that found factors other than radiographic disease severity as the most important determinants of TKA use [13]. This is highlighted by the study by Dieppe et al., that examined 1327 cases of hip OA and found no relationship between the radiographic severity of OA and reported levels of disability [34]. Our study did not assess the use of disease or symptom modifying agents, including bracing, injections, therapy or analgesic and anti-inflammatory medications. Additionally, preclusive socio-economic variables for the decision to undergo TKA were not assessed.

Previous examinations into determinants of TKA use have cited differences in access to care [47]. In our study, all subjects sought care from a single orthopedic surgeon for management of knee pain prior to functional evaluation. Patients were aware of the surgical and non-surgical options available to them and all were from the local area, referred to the physical therapy clinic from a single orthopedic surgeon who does not perform total knee replacements. Additionally, the sample was a relatively homogeneous group from a narrow geographical window. Differences in access to care and socio-economic status were not likely to have been determinants for these participants. This may also explain why no differences were found between males and females in this study. All subjects were well aware of the option of TKA for management of the disease and were not likely biased by any surgeon preference.

## Conclusions

The results of this study (1) add to the understanding of functional motivations for undergoing TKA in persons with end-stage knee OA and (2) define potentially modifiable risk factors that should be the target of pre-operative interventions in persons who wish to delay TKA. Younger patients with full knee ROM who have a higher self-perception of function are less likely to undergo TKA. Physicians and clinicians should be aware that potentially modifiable factors, such as knee ROM, can be addressed to potentially postpone the need for TKA. Functional determinants of TKA use may play an important role in a sample with equal access to healthcare. Future work should address functional determinants in a larger sample with significant disparity in socio-economic variables. Future longitudinal interventional studies addressing ROM and KOS-ADLS or age-related impairments are required to determine if modifying risk factors in persons with end-stage knee OA will reduce or delay TKA use. The Delaware Osteoarthritis Profile is an easily administered set of clinical tests that can provide important information about the likelihood of future TKA.

## Competing interests

The authors declare that they have no competing interests.

## Authors' contributions

JZ carried out the statistical analysis and interpretation of the data and was responsible for drafting of the manuscript. LSM was responsible for the design and conception of the project, oversaw data collections and contributed to the comprehensive editing and review of the manuscript. MJA was responsible for screening all of the patients for inclusion in the study and contributed to the study design and conception. All authors read and approved of the final version of the manuscript.

## Pre-publication history

The pre-publication history for this paper can be accessed here:

http://www.biomedcentral.com/1471-2474/11/86/prepub

## References

[B1] Guccione AA Felson DT Anderson JJ Anthony JM Zhang Y Wilson PW Kelly-Hayes M Wolf PA Kreger BE Kannel WB The effects of specific medical conditions on the functional limitations of elders in the Framingham Study Am J Public Health 1994 84 3 351 358 10.2105/AJPH.84.3.351 8129049 PMC1614827

[B2] Oliveria SA Felson DT Reed JI Cirillo PA Walker AM Incidence of symptomatic hand, hip, and knee osteoarthritis among patients in a health maintenance organization Arthritis and rheumatism 1995 38 8 1134 1141 10.1002/art.1780380817 7639811

[B3] Jacobs J Andersson G Bell J Weinstein S The Burden of Musculoskeletal Diseases in the United States - Executive Statement Bone and Joint Decade 2008 Rosemont: AAOS

[B4] Dieppe P Basler HD Chard J Croft P Dixon J Hurley M Lohmander S Raspe H Knee replacement surgery for osteoarthritis: effectiveness, practice variations, indications and possible determinants of utilization Rheumatology (Oxford, England) 1999 38 1 73 83 10.1093/rheumatology/38.1.73 10334686

[B5] NIH NIH Consensus Statement on total knee replacement NIH Consens State Sci Statements 2003 20 1 1 34 17308549

[B6] Dieppe P Management of osteoarthritis of the hip and knee joints Current opinion in rheumatology 1993 5 4 487 493 10.1097/00002281-199305040-00014 8357745

[B7] Gossec L Hawker G Davis AM Maillefert JF Lohmander LS Altman R Cibere J Conaghan PG Hochberg MC Jordan JM OMERACT/OARSI initiative to define states of severity and indication for joint replacement in hip and knee osteoarthritis The Journal of rheumatology 2007 34 6 1432 1435 17552070

[B8] Mancuso CA Ranawat CS Esdaile JM Johanson NA Charlson ME Indications for total hip and total knee arthroplasties. Results of orthopaedic surveys The Journal of arthroplasty 1996 11 1 34 46 10.1016/S0883-5403(96)80159-8 8676117

[B9] Maillefert JF Roy C Cadet C Nizard R Berdah L Ravaud P Factors influencing surgeons' decisions in the indication for total joint replacement in hip osteoarthritis in real life Arthritis and rheumatism 2008 59 2 255 262 10.1002/art.23331 18240195

[B10] Borkhoff CM Hawker GA Kreder HJ Glazier RH Mahomed NN Wright JG The effect of patients' sex on physicians' recommendations for total knee arthroplasty Cmaj 2008 178 6 681 687 18332383 10.1503/cmaj.071168 PMC2263116

[B11] Borkhoff CM Hawker GA Kreder HJ Glazier RH Mahomed NN Wright JG Patients' gender affected physicians' clinical decisions when presented with standardized patients but not for matching paper patients Journal of clinical epidemiology 2009 62 5 527 541 10.1016/j.jclinepi.2008.03.009 19348978

[B12] Hawker GA Wright JG Coyte PC Williams JI Harvey B Glazier R Wilkins A Badley EM Determining the need for hip and knee arthroplasty: the role of clinical severity and patients' preferences Medical care 2001 39 3 206 216 10.1097/00005650-200103000-00002 11242316

[B13] Hawker GA Guan J Croxford R Coyte PC Glazier RH Harvey BJ Wright JG Williams JI Badley EM A prospective population-based study of the predictors of undergoing total joint arthroplasty Arthritis and rheumatism 2006 54 10 3212 3220 10.1002/art.22146 17009255

[B14] Wittink H Rogers W Sukiennik A Carr DB Physical functioning: self-report and performance measures are related but distinct Spine 2003 28 20 2407 2413 10.1097/01.BRS.0000085304.01483.17 14560092

[B15] Hidding A van Santen M De Klerk E Gielen X Boers M Geenen R Vlaeyen J Kester A Linden S van der Comparison between self-report measures and clinical observations of functional disability in ankylosing spondylitis, rheumatoid arthritis and fibromyalgia The Journal of rheumatology 1994 21 5 818 823 8064720

[B16] Petterson SC Raisis L Bodenstab A Snyder-Mackler L Disease-specific gender differences among total knee arthroplasty candidates The Journal of bone and joint surgery 2007 89 11 2327 2333 10.2106/JBJS.F.01144 17974873

[B17] Hawker GA Wright JG Coyte PC Williams JI Harvey B Glazier R Badley EM Differences between men and women in the rate of use of hip and knee arthroplasty The New England journal of medicine 2000 342 14 1016 1022 10.1056/NEJM200004063421405 10749964

[B18] Katz BP Freund DA Heck DA Dittus RS Paul JE Wright J Coyte P Holleman E Hawker G Demographic variation in the rate of knee replacement: a multi-year analysis Health services research 1996 31 2 125 140 8675435 PMC1070109

[B19] Dunlop DD Song J Manheim LM Chang RW Racial disparities in joint replacement use among older adults Medical care 2003 41 2 288 298 10.1097/00005650-200302000-00010 12555056

[B20] McAlindon TE Cooper C Kirwan JR Dieppe PA Determinants of disability in osteoarthritis of the knee Annals of the rheumatic diseases 1993 52 4 258 262 10.1136/ard.52.4.258 8484690 PMC1005622

[B21] Gossec L Jordan JM Lam MA Fang F Renner JB Davis A Hawker GA Dougados M Maillefert JF Comparative evaluation of three semi-quantitative radiographic grading techniques for hip osteoarthritis in terms of validity and reproducibility in 1404 radiographs: report of the OARSI-OMERACT Task Force Osteoarthritis and cartilage/OARS, Osteoarthritis Research Society 2009 17 2 182 187 10.1016/j.joca.2008.06.009 18691910

[B22] Cicuttini FM Jones G Forbes A Wluka AE Rate of cartilage loss at two years predicts subsequent total knee arthroplasty: a prospective study Annals of the rheumatic diseases 2004 63 9 1124 1127 10.1136/ard.2004.021253 15115714 PMC1755122

[B23] Toivanen AT Arokoski JP Manninen PS Heliovaara M Haara MM Tyrvainen E Niemitukia L Kroger H Agreement between clinical and radiological methods of diagnosing knee osteoarthritis Scand J Rheumatol 2007 36 1 58 63 10.1080/03009740600759886 17454937

[B24] Gossec L Tubach F Baron G Ravaud P Logeart I Dougados M Predictive factors of total hip replacement due to primary osteoarthritis: a prospective 2 year study of 505 patients Annals of the rheumatic diseases 2005 64 7 1028 1032 10.1136/ard.2004.029546 15640268 PMC1755580

[B25] Parent E Moffet H Preoperative predictors of locomotor ability two months after total knee arthroplasty for severe osteoarthritis Arthritis and rheumatism 2003 49 1 36 50 10.1002/art.10906 12579592

[B26] Mizner RL Petterson SC Stevens JE Axe MJ Snyder-Mackler L Preoperative quadriceps strength predicts functional ability one year after total knee arthroplasty The Journal of rheumatology 2005 32 8 1533 1539 16078331

[B27] March L Cross M Tribe K Lapsley H Courtenay B Brooks P Cost of joint replacement surgery for osteoarthritis: the patients' perspective The Journal of rheumatology 2002 29 5 1006 1014 12022316

[B28] Kellgren JH Lawrence JS Radiological assessment of osteo-arthrosis Annals of the rheumatic diseases 1957 16 4 494 502 10.1136/ard.16.4.494 13498604 PMC1006995

[B29] Mizner RL Petterson SC Snyder-Mackler L Quadriceps strength and the time course of functional recovery after total knee arthroplasty J Orthop Sports Phys Ther 2005 35 7 424 436 16108583 10.2519/jospt.2005.35.7.424

[B30] Cibere J Bellamy N Thorne A Esdaile JM McGorm KJ Chalmers A Huang S Peloso P Shojania K Singer J Reliability of the knee examination in osteoarthritis: effect of standardization Arthritis and rheumatism 2004 50 2 458 468 10.1002/art.20025 14872488

[B31] Irrgang JJ Snyder-Mackler L Wainner RS Fu FH Harner CD Development of a patient-reported measure of function of the knee The Journal of bone and joint surgery 1998 80 8 1132 1145 9730122 10.2106/00004623-199808000-00006

[B32] Marx RG Jones EC Allen AA Altchek DW O'Brien SJ Rodeo SA Williams RJ Warren RF Wickiewicz TL Reliability, validity, and responsiveness of four knee outcome scales for athletic patients The Journal of bone and joint surgery 2001 83-A 10 1459 1469 11679594 10.2106/00004623-200110000-00001

[B33] Kennedy DM Stratford PW Wessel J Gollish JD Penney D Assessing stability and change of four performance measures: a longitudinal study evaluating outcome following total hip and knee arthroplasty BMC musculoskeletal disorders 2005 6 3 10.1186/1471-2474-6-3 15679884 PMC549207

[B34] Dieppe P Judge A Williams S Ikwueke I Guenther KP Floeren M Huber J Ingvarsson T Learmonth I Lohmander LS Variations in the pre-operative status of patients coming to primary hip replacement for osteoarthritis in European orthopaedic centres BMC musculoskeletal disorders 2009 10 19 10.1186/1471-2474-10-19 19208230 PMC2654855

[B35] Manninen P Riihimaki H Heliovaara M Makela P Overweight, gender and knee osteoarthritis Int J Obes Relat Metab Disord 1996 20 6 595 597 8782738

[B36] Felson DT Anderson JJ Naimark A Walker AM Meenan RF Obesity and knee osteoarthritis. The Framingham Study Annals of internal medicine 1988 109 1 18 24 3377350 10.7326/0003-4819-109-1-18

[B37] Shelbourne KD Gray T Minimum 10-year results after anterior cruciate ligament reconstruction: how the loss of normal knee motion compounds other factors related to the development of osteoarthritis after surgery Am J Sports Med 2009 37 3 471 480 10.1177/0363546508326709 19059893

[B38] Roe J Pinczewski LA Russell VJ Salmon LJ Kawamata T Chew M A 7-year follow-up of patellar tendon and hamstring tendon grafts for arthroscopic anterior cruciate ligament reconstruction: differences and similarities Am J Sports Med 2005 33 9 1337 1345 10.1177/0363546504274145 16002487

[B39] Gensburger D Arlot M Sornay-Rendu E Roux JP Delmas P Radiologic assessment of age-related knee joint space changes in women: A 4-year longitudinal study Arthritis and rheumatism 2009 61 3 336 343 10.1002/art.24342 19248124

[B40] Deyle GD Henderson NE Matekel RL Ryder MG Garber MB Allison SC Effectiveness of manual physical therapy and exercise in osteoarthritis of the knee. A randomized, controlled trial Annals of internal medicine 2000 132 3 173 181 10651597 10.7326/0003-4819-132-3-200002010-00002

[B41] Deyle GD Allison SC Matekel RL Ryder MG Stang JM Gohdes DD Hutton JP Henderson NE Garber MB Physical therapy treatment effectiveness for osteoarthritis of the knee: a randomized comparison of supervised clinical exercise and manual therapy procedures versus a home exercise program Physical therapy 2005 85 12 1301 1317 16305269

[B42] Fransen M Crosbie J Edmonds J Physical therapy is effective for patients with osteoarthritis of the knee: a randomized controlled clinical trial The Journal of rheumatology 2001 28 1 156 164 11196518

[B43] Baker KR Nelson ME Felson DT Layne JE Sarno R Roubenoff R The efficacy of home based progressive strength training in older adults with knee osteoarthritis: a randomized controlled trial The Journal of rheumatology 2001 28 7 1655 1665 11469475

[B44] Fransen M McConnell S Land-based exercise for osteoarthritis of the knee: a metaanalysis of randomized controlled trials The Journal of rheumatology 2009 36 6 1109 1117 10.3899/jrheum.090058 19447940

[B45] Fransen M McConnell S Bell M Therapeutic exercise for people with osteoarthritis of the hip or knee. A systematic review The Journal of rheumatology 2002 29 8 1737 1745 12180738

[B46] Maly MR Costigan PA Olney SJ Determinants of self efficacy for physical tasks in people with knee osteoarthritis Arthritis and rheumatism 2006 55 1 94 101 10.1002/art.21701 16463419

[B47] Skinner J Weinstein JN Sporer SM Wennberg JE Racial, ethnic, and geographic disparities in rates of knee arthroplasty among Medicare patients The New England journal of medicine 2003 349 14 1350 1359 10.1056/NEJMsa021569 14523144

